# Current and New Novel Combination Treatments for Metastatic Triple-Negative Breast Cancer

**DOI:** 10.3390/curroncol29070377

**Published:** 2022-07-07

**Authors:** Mehrnoosh Pauls, Stephen Chia, Nathalie LeVasseur

**Affiliations:** BC Cancer Vancouver Centre, University of British Columbia, 600 W 10th Ave, Vancouver, BC V5Z 4E6, Canada; schia@bccancer.bc.ca

**Keywords:** metastatic TNBC, immunotherapy, PARP inhibitor, antibody-drug conjugate (ADC), PI3K/AKT/mTOR

## Abstract

Triple-negative breast cancer (TNBC) has a worse prognosis and remains the most challenging breast cancer subtype to treat. This is largely related to the heterogeneity of this disease and the lack of reliable oncological targets. In this review, we discuss the current standard-of-care treatment options for metastatic TNBC, including recent advances with the use of immunotherapy, PARP inhibitors and antibody-drug conjugates. This review also explores new agents and novel combinations arising in the field for the treatment of advanced TNBC.

## 1. Background

Triple-negative breast cancer (TNBC) remains a challenging disease to treat due to its heterogeneity and poor prognosis compared to hormone receptor (HR)-positive and human epidermal growth factor receptor 2 (HER2)-positive breast cancers [1]. Patients with TNBC who relapse often do so within the first 3 years following diagnosis and frequently develop visceral metastases, with the minority not surviving beyond 2 years [2]. While TNBC has traditionally been defined as the absence of oestrogen receptors, progesterone receptor staining on immunohistochemistry (IHC) and a negative HER2 test via IHC and/or in situ hybridization (ISH), it is well recognized that TNBC comprises multiple histomolecular subtypes. A study conducted by Lehmann et al. analysed gene expression profiles from 578 TNBC cases and grouped them into six different molecular subtypes, namely the basal-like 1 (BL1), basal-like 2 (BL2), immunomodulatory (IM), mesenchymal (M), mesenchymal stem-like (MSL), and luminal androgen receptor (LAR), supporting the paradigm of heterogeneity [3]. However, the use of targeted agents remains challenging given the absence of reliable oncological targets beyond ER, HER2 and BRCA. Recent advances in the treatment of TNBC have harnessed the interplay between the tumour microenvironment and immunogenicity along with traditionally recognized histological features and molecular alterations. This review discusses the current standard-of-care treatment paradigms in the management of metastatic TNBC based on drug class and discusses novel drug combinations that are currently under study.

### 1.1. Current Management of Metastatic Breast Cancer

**Figure 1:**
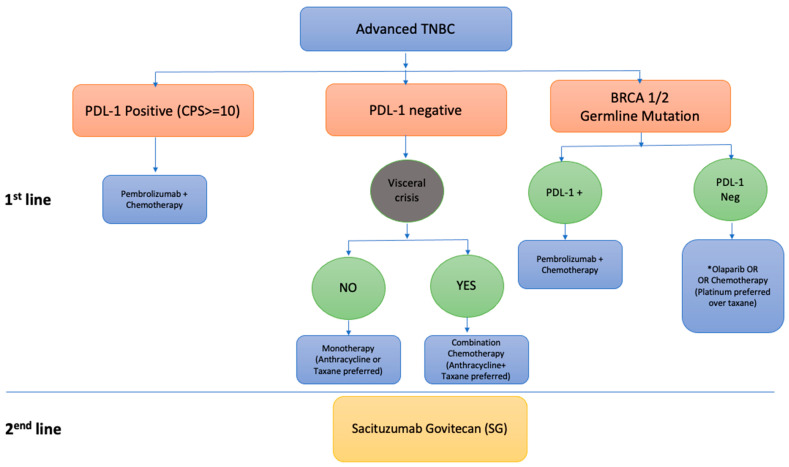
Summarized algorithm for treatment for mTNBC based on current phase III evidence available. * This recommendation is based on meaningful OS among the prespecified subgroup that received olaparib as first-line treatment who had not received prior chemotherapy (HR 0.51, 95% CI 0.29–0.90) in OlympiAD trial.

Despite the development of targeted agents for the treatment of HR-positive and HER2-positive breast cancers, cytotoxic chemotherapy remains integral to the treatment of breast cancer, particularly triple-negative breast cancer. However, notable advancements have recently been made with the use of immunotherapy, PARP inhibitors and antibody-drug conjugates (ADCs), thus expanding available treatment options for patients with advanced TNBC. Results from the most recent landmark, practice-changing studies are detailed below and summarized in Table 1. A proposed management algorithm for patients with advanced TNBC is presented in Figure 1.

### 1.2. Immunotherapy

Breast cancer has historically been thought of as an immune cold disease [4]. However, more recent studies have suggested some sensitivity to immunotherapy in certain TNBC subtypes, bringing this to the forefront in recent clinical trials [5,6]. Particular characteristics that have been predictive of immunotherapy benefit in TNBC include a high level of programmed cell death ligand 1 (PD-L1) expression and more tumour infiltration lymphocytes (TILs) in contrast to other breast cancer subtypes. In addition, many TNBCs have a higher tumour mutational burden, which has been associated with potential immunotherapy benefit, though most of the data stem from other tumour types and have been extrapolated to breast cancer [4].

This preclinical and translational work led to the investigation of immune checkpoint inhibitors in TNBC. Two drugs, pembrolizumab and atezolizumab, have been studied in phase III trials in the metastatic TNBC population [5,6,7]. The first of these, Impassion 130, studied the role of atezolizumab (a PD-L1 inhibitor) in combination with nab-paclitaxel for unresectable, locally advanced or metastatic TNBC patients who had not received prior therapy in the metastatic setting [6]. After 12.9 months of follow up, the intention-to-treat population showed a modest improvement in median progression-free survival (PFS) of 7.2 months for the atezolizumab and nab-paclitaxel group compared to 5.5 months with the placebo and nab-paclitaxel (HR 0.80, 95% CI 0.69–0.92). There was also modest numerical improvement in the median overall survival (OS) of 21.3 months in the atezolizumab and nab-paclitaxel group compared to 17.6 months with the placebo and nab-paclitaxel (HR 0.84, 95% CI 0.69–1.02). While this was a deviation from the original statistical plan, both the PFS and OS were significantly higher among PDL-1-positive tumours defined as PDL-1 staining ≥1% of tumour-infiltrating immune cells on immunohistochemical testing with the SP142 companion assay. Among the PDL-1-positive group, the median PFS was 7.5 months with atezolizumab and nab-paclitaxel vs. 5.0 months with the placebo and nab-paclitaxel (HR 0.62, 95% CI 0.49–0.78), and the median OS was 25 months vs. 15.5 months, which was descriptively in favour of atezolizumab and nab-paclitaxel (HR 0.62, 95% CI 0.45–0.86), which was maintained at the final OS analysis [6,8]. This led to accelerated approval by the FDA to use a combination of atezolizumab and nab-paclitaxel in metastatic TNBC. However, it was later withdrawn following the publication of Impassion 131, which looked at the combination of atezolizumab and solvent-based paclitaxel compared to a placebo and solvent-based paclitaxel. Despite a similar design, this study failed to show an improvement in PFS or OS among the intention-to-treat population and the PDL-1-positive population, with in fact a trend for worse survival for the experimental arm [7]. Reasons postulated to explain this lack of benefit included the choice of the chemotherapy partner, the effect on the tumour microenvironment and the use of steroids, amongst others.

Subsequently, the results of KEYNOTE 355 were presented, which was a phase III trial studying the role of pembrolizumab (a PD-1 inhibitor) in combination with chemotherapy (paclitaxel, nab-paclitaxel or carboplatin and gemcitabine) among individuals with locally advanced inoperable or metastatic TNBC who had a greater or equal to 6-month disease-free survival interval [5]. PD-L1 expression was established using the 22C3 pharmDX companion assay to establish the combined positive score (CPS), representing the percentage of tumour cells and immune cells with PDL1 staining. This trial demonstrated a statistically significant improvement in PFS of 9.7 months in the pembrolizumab and chemotherapy group compared to 5.6 months in the placebo and chemotherapy group among the subgroup with PD-L1-positive disease with CPS ≥ 10 (HR 0.65, 95% CI 0.49–0.86). The overall survival among PD-L1-positive patients was 23 months in the pembrolizumab and chemotherapy group vs. 16 months in the placebo and chemotherapy group (HR 0.73, 95% CI 0.55–0.95) [9]. This led to the FDA approval of pembrolizumab combined with chemotherapy for metastatic TNBC with PDL-1-positive disease CPS ≥ 10, which is now acknowledged as the standard of care as the first-line therapy in patients with PD-L1 positivity with CPS ≥ 10. It is important to note that pembrolizumab monotherapy without chemotherapy did not show significant improvements in OS regardless of PD-L1 status, thus reinforcing the importance of administering pembrolizumab in combination with upfront chemotherapy [10].

### 1.3. PARP Inhibitors

The incidence of breast cancer susceptibility gene (BRCA) mutations is as high as approximately 20% among TNBC patients, making it one of the most common genetic alterations seen in this patient population [11]. BRCA mutations lead to a defect in homologous recombination (HR) repair, which is necessary to repair double-stranded DNA breaks. Poly ADP ribose polymerase (PARP) inhibitors exploit this homologous recombination repair deficiency (HRD) with the inability to repair single-strand DNA breaks that are converted to double-stranded DNA breaks during replication in BRCA1/2-deficient tumours, ultimately leading to synthetic lethality [12].

Several phase III trials, including OlympiAD, EMBRACA and BROCADE 3, have studied the role of the PARP inhibitors in TNBC patients with BRCA1/2 germline mutations [13,14,15]. OlympiAD was a phase III clinical trial comparing olaparib to physicians’ choices of chemotherapy among HER-2-negative metastatic breast cancer patients with germline BRCA mutations who received no more than two lines of prior therapy [13]. This trial showed statistically significant results with a median PFS of 7.0 months with olaparib and 4.2 months among the chemotherapy group (HR 0.58, 95% CI 0.43–0.80). In addition, olaparib was better tolerated than chemotherapy with fewer grade 3 adverse events [13]. However, there was no significant improvement in OS between the two groups after 25 months of follow up with a median OS of 19.3 months with olaparib and 17.1 months with chemotherapy (HR 0.91, 95% CI 0.66–1.23) [16]. There was, however, a possibly meaningful OS benefit among the prespecified subgroup that received olaparib as a first-line treatment who had not received prior chemotherapy (HR 0.51, 95% CI 0.29–0.90) [16].

EMBRACA is another phase III clinical trial that compared the PARP inhibitor, Talazoparib, to physicians’ choices of chemotherapy among advanced HER-2-negative breast cancer patients with a germline BRCA mutation who had received no more than three previous lines of treatment [14]. This trial also demonstrated significant improvements in the median PFS of 8.6 months with talazoparib compared to 5.6 months with chemotherapy (HR 0.54, 95% CI 0.41–0.71). There was also a better objective response rate with talazoparib (62.6%) in contrast to chemotherapy (27.2%). In addition, the quality of life was better for the arm that received talazoparib [14]. However, similar to the OlympiAD study, the EMBRACA trial did not significantly improve the OS (median OS 19.3 months in the talazoparib group and 19.5 months for the chemotherapy group after 44.9 months and 36.8 months of follow up, respectively) (HR 0.84, 95% CI 0.67–1.07) [17].

It is essential to note that the comparator arms in both the OlympiAD and EMBRACA studies comprised cytotoxic chemotherapy drugs that did not include anthracyclines, taxanes or platinum drugs, which are often used as first-line treatments for metastatic TNBC. In the OlympiAD study, physicians’ choices of chemotherapy included capecitabine, eribulin or vinorelbine [13]. In the EMBRACA study, physicians’ chemotherapy choices included gemcitabine, capecitabine, eribulin or vinorelbine [14]. Therefore, it remains unclear how PARP inhibitors may compare to first-line anthracyclines, taxanes or platinum drugs in the advanced setting. A subset of 121 patients with metastatic TNBC from the OlympiAD study did reveal an improved PFS relative to the treatment of the physician’s choice for patients who had received anthracycline and taxane in the adjuvant or advanced settings (HR 0.43, 95% CI 0.29–0.63). This is particularly relevant as an OS advantage was noted for patients who received olaparib as a first-line treatment and had not received prior chemotherapy in the OlympiAD study, as previously discussed [16].

Finally, another relevant phase III study of PARP inhibitors is the BROCADE 3 trial, which evaluated the role of veliparib or placebo in combination with a platinum doublet (carboplatin–paclitaxel) in patients with advanced HER-2-negative breast cancer with a germline BRCA mutation who had received no more than two previous lines of cytotoxic therapy for metastatic breast cancer [15]. This study demonstrated a numerical improvement in PFS after 35 months of follow up. The median PFS was 14.5 months in the veliparib plus platinum doublet combination group and 12.6 months in the placebo plus platinum doublet combination group (HR 0.71, 95% CI 0.57–0.88) [15]. The study met its primary endpoint, which was PFS and the tolerability of PARP inhibitors in combination with chemotherapy. The data regarding overall survival are not mature yet and are expected to be reported in the near future. In current clinical practice, PARP inhibitors are approved based on these three critical trials for patients with metastatic breast cancer with a germline BRCA1/2 mutation.

### 1.4. Antibody-Drug Conjugates

Epithelial cells commonly express trophoblast cell-surface antigen 2 (Trop-2), and its overexpression in cancer cells is generally associated with worse prognosis and survival outcomes [18]. Antibody-drug conjugates have shown promising results in advanced TNBC. Sacituzumab govitecan (SG) is a novel ADC that works by binding Trop-2 which is highly expressed in the majority of TNBCs and delivers the SN-38 payload drug to the cancer cells with a hydrolysable linker [19]. SN-38, an active metabolite of irinotecan, reversibly binds the topoisomerase 1 cleavage complex on DNA and interrupts DNA replication in cancer cells, causing S-phase-specific cell death [18].

SG was initially evaluated in a basket trial in patients with epithelial cancers who had failed conventional treatments [20]. In the trial, a subgroup of patients with mTNBC had a significant objective response to this drug with a median progression-free survival (PFS) of 5.5 months and overall survival (OS) of 13 months. This clinical trial led to the approval of SG in the U.S. and the initiation of the phase III ASCENT trial. The ASCENT trial was a phase III clinical trial that evaluated SG compared to single-agent chemotherapy among metastatic TNBC patients with refractory disease after two or more lines of treatment [19]. This trial showed a benefit among the group that received SG compared to the chemotherapy group with an improved median PFS of 5.6 vs.1.7 months (HR 0.41, 95% CI 0.32–0.52) and a median OS of 12.1 vs. 6.7 months (HR 0.48, 95% CI 0.38–0.59), respectively [19]. There were adverse events associated with SG, with the most common grade 3–4 side effects being neutropenia and diarrhoea, though these were manageable with supportive care and patient education [19]. As a result of the Ascent trial, SG is the first ADC approved for use in patients with refractory TNBC after two or more lines of prior treatment.

## 2. Emerging Drugs and Novel Combinations for TNBC

### 2.1. Exploring Combinations with Antibody-Drug Conjugates (ADCs)

In the past two decades, ADCs have emerged as an attractive means to treat breast cancer, which is of great interest in TNBC given the paucity of actionable molecular targets with tangible clinical benefit. ADCs comprise three primary components, including an antibody directed to the tumour antigen, a linker and a cytotoxic payload [21]. Minor changes in ADCs’ components can lead to significant changes in the therapeutic index by exploiting the characteristics and interplay of each component, as well as their interactions with the tumour and the microenvironment. Currently, sacituzumab govitecan (SG) is the only ADC approved for the treatment of metastatic TNBC, as previously discussed in this review [19]. However, more than 60 ADCs are at different stages of development for use in breast cancer patients, including several trials for TNBC, with molecular targets such as TROP-2, LIV-1, HER2, HER3 and ROR2, amongst others. The selected agents included in this review are trastuzumab deruxtecan (T-DXd) (NCT04556773), ladiratuzumab vedotin (SGN-LIV1a) (NCT03310957), vic-trastuzumab duocarmazine (SYD985) (NCT04602117), anti-B7-H3-ADC (MGC018) (NCT03729596), CAB-ROR-ADC (BA3021) (NCT03504488), datopotamab deruxtecan (DS-1062) (NCT05374512) and tusamitamab Ravtansine (SAR408701) (NCT04659603) [22] (Table 2).

### 2.2. ADCs and Chemotherapy

Transtuzumab deruxtecan (T-DXd) is another novel ADC composed of a topoisomerase I inhibitor deruxtecan linked by a cleavable peptide-based linker to a humanized antibody against HER-2. Currently, T-DXd has been approved for usage as second-line anti-HER-2-directed therapy for metastatic HER-2-positive disease in the clinical setting based on the Destiny-Breast-03 phase III trial, showing significant improvement in PFS (HR 0.28, 95% CI 0.22–0.37) and OS (HR 0.55, 95% CI 0.36–0.86) when compared to trastuzumab emtansine (T-DM1) [23]. However, T-DXd has potential as a treatment agent beyond HER-2-positive disease. In the first dose-escalation phase of the phase I study of T-DXd, 8 out of 24 patients had low-HER-2-expressing tumours with either immunohistochemistry (IHC) 1+ or IHC 2+ /ISH negative [24]. Even though most responders were in the high-HER-2 group, defined as IHC 3+, two patients with low-HER-2-expressing tumours were also responders with T-DXd [24]. This ultimately led to the investigation of T-DXd among HER-2 low-expressing breast cancers, including TNBC. DESTINY-Breast-04 is a large multicentre phase III trial that has evaluated the role of T-DXd compared to physicians’ choices of chemotherapy among unresectable and/or metastatic HER-2 low-expressing TNBC patients (NCT03734029) [25]. Early results released in a press release state the study met its primary endpoint of improved PFS, as well as demonstrating statistically significant improvements in OS. There are also clinical trials evaluating novel combinations with T-DXd. The DESTINY-Breast08 is a large trial that investigates dose-finding, dose-expansion, safety, tolerability, pharmacokinetics and anti-tumour activity of T-DXd in combination with multiple other anti-cancer agents including chemotherapy, immunochemotherapy, AKT inhibitors, and other drugs among metastatic HER2-low breast cancer cases, including TNBC patients (NCT04556773) [26]. ISPY-P1.01 is another phase I trial that is currently evaluating the safety of another novel ADC vic-trastuzumab duocarmazine (SYD985), combined with weekly paclitaxel chemotherapy among patients with metastatic breast cancer, including TNBC (NCT04602117) [27].

### 2.3. ADCs and Immunotherapy

Ladiratuzumab vedotin (SGN-LIV1a) is a novel ADC composed of a monomethyl auristatin E (MMAE) microtubule-disrupting agent linked via a protease-cleavable linker to humanized anti–LIV-1 igG1 monoclonal antibody [28]. This novel ADC functions by delivering MMAE to cells that express LIV-1, leading to microtubule disruption causing cell cycle arrest and cell death. Furthermore, it is hypothesized that SGN-LIV1a drives immune response via immunogenic cell death (ICD), suggesting a synergistic effect when combined with immunotherapy by creating a favourable microenvironment [28]. LIV-1 was previously studied in HR-positive tumours and subsequently was found to be upregulated in TNBC. Early-phase studies have shown promising anti-tumour activity in heavily treated metastatic TNBC. In a phase I trial dose-escalation and expansion cohort, the safety and anti-tumour activity of SGN-LIV1a were evaluated among 44 locally advanced or metastatic TNBCs [29]. Their results were encouraging, showing an ORR of 32%, disease control rate (DCR) of 64% and CBR of 36% among 16 heavily treated advanced TNBC patients. The median PFS was 11.3 weeks (95% CI 6.1–17.1), with ten patients remaining on treatment [29]. These early phases led to openings of other studies investigating SGN-LIV1a as a therapeutic agent among advanced TNBC patients both as monotherapy and combination therapy. SGNLVA-002 is a phase IB/II clinical trial looking at the safety and efficacy of SGN-LIV1A in combination with pembrolizumab (PD-1 inhibitor) as a first-line treatment for patients with unresectable locally advanced or metastatic TNBC (NCT03310957) [30].

Morpheus-TNBC is another large phase IB/II clinical trial that is studying the role of multiple immunotherapy-based treatment combinations among patients with inoperable, locally advanced or metastatic TNBC. In this trial, atezolizumab (PD-L1 inhibitor) is the immunotherapeutic agent combined with anti-neoplastic agents such as chemotherapy, AKT inhibitors, VEGF inhibitors, monoclonal antibodies, and ADCs. The novel combination of ADC and immunotherapy in this trial is atezolizumab combined with SGN-LIV1A and atezolizumab combined with SG. The primary endpoint of this trial is the ORR and rates of AE. Secondary endpoints include PFS, disease control rate (DCR), duration of response (DOR) and OS at 12 months, 18 months and 5 years (NCT03424005) [31].

Furthermore, there are also other phase I/II trials that are investigating the role of other novel ADC agents in combination with PD-1 inhibitor summarized in Table 2 (NCT03729596, NCT03504488 and NCT04468061) [32,33,34].

### 2.4. ADCs and PARP Inhibitors

The ASCENT trial demonstrated improvements in PFS and OS among metastatic TNBC cases treated with the use of SG [19]. Further, the EMBARACA trial demonstrated that talazoparib improved the PFS and ORR among HER-2-negative breast cancer patients with germline BRCA mutations. It has been hypothesized that a combination of SG and talazoparib could have synergic effects by halting cancer growth and proliferation via SG, thus delivering a payload that would ultimately interrupt DNA replication and DNA repair. This combination is currently under investigation in a phase IB/II clinical trial in metastatic TNBC patient populations. The primary objective is to evaluate dose-limiting toxicity, and the secondary objective is to evaluate the time to tumour response, duration of response, PFS and OS (NCT04039230) [35]. The estimated completion timeline for this trial is October 2024, which could provide the rationale for a phase III trial and possibly pave the way for additional combinations of ADCs with PARP inhibitors.

### 2.5. ADCs and PI3K Inhibitors

The development and targeting of the proto-oncogenic phosphatidylinositol-3-kinase (PI3K)/protein kinase B/mammalian target of rapamycin (PI3K/AKT/mTOR) pathway in TNBC is evolving. Alpelisib is a PI3K inhibitor that was initially approved in combination with fulvestrant for use among metastatic HR-positive, HER-2-negative breast cancer patients, based on results of the SOLAR-1 trial, though PI3K-activating mutations are also present in 8–25% of TNBC cases [36,37]. In addition, the loss of the phosphatase and tensin homologue deleted chromosome 10 (PTEN) function or reduced expression, leading to the hyperactivation of the PI3K signalling pathway, which occurs frequently in TNBC [37]. As a result, there has been interest in evaluating the role of various alpelisib combinations as a treatment option for TNBC. One such combination is evaluating the role of an ADC in combination with a PI3K inhibitor. The rationale is to interrupt DNA replication with SG and exploit potential synergy with an agent targeting the PI3K/AKT/mTOR pathway needed for cancer cell survival, proliferation and invasion. This is postulated to decrease cancer cell survival. ASSET is a phase I clinical trial evaluating the role of alpelisib in combination with SG in metastatic breast cancer patients, including metastatic TNBC. The study’s primary endpoint is finding a recommended phase II dose (RP2D). The secondary endpoint is to evaluate the pharmacokinetics of the combination and ORR (NCT05143229) [38].

Despite the rapidly growing interest in ADCs and the potential for the greater personalization of treatment and enhanced bystander effect, we await confirmatory data of activity for the agents discussed in this review. As a cautionary tale, we cite the example of glembatumumab vedotin, which initially showed an ORR of 18% compared to 0% with the investigator’s choice of chemotherapy among TNBC cases and 40% compared to 0% ORR among glycoprotein NMB (gpNMB)-overexpressing TNBC cases [39]. This paved the way for the METRIC phase II study, which failed to show an improvement in ORR, PFS and OS among metastatic gpNMB over-expressing TNBC cases [40].

### 2.6. PI3K/AKT/mTOR Targeted Agents

The PI3K/AKT/mTOR signalling pathway plays a crucial role in oncogenesis by promoting cancer cell survival, proliferation motility and invasion. PTEN also plays a vital role as a tumour suppressor via the negative regulation of the PI3K/AKT/mTOR signalling pathway [41,42]. The activation of the PI3K/AKT/mTOR signalling pathway or loss of PTEN tumour suppression is common in TNBC, making this pathway an attractive target for therapy [42,43]. Ipatasertib is an AKT inhibitor studied in TNBC in the phase II LOTUS trial [44]. This trial investigated the addition of ipatasertib to paclitaxel compared to a placebo and paclitaxel as a first-line treatment amongst 124 metastatic or locally advanced TNBC patients. The results demonstrated an improvement in the median PFS after 10 months of follow up, 6.2 months with the AKT inhibitor and paclitaxel combination and 4.9 months with the paclitaxel placebo (HR 0.60, 95% CI 0.37–0.98) [44]. The PFS difference was more pronounced among the PI3K/AKT/PTEN-altered group (HR 0.44, 95% CI 0.20–0.99). The median overall survival (OS) results numerically favoured the AKT inhibitor combination group compared to the placebo (25.8 months and 16.9 months, respectively, HR 0.80, 95% CI 0.50–1.28), though this was not statistically significant [45]. These results led to other clinical trials studying AKT inhibitors combined with chemotherapy or immunotherapy as therapeutic options for metastatic TNBC. IPATunity130 (NCT03337724) was a phase III trial evaluating the efficacy of the combination of ipatasertib and paclitaxel compared to a placebo and paclitaxel in locally advanced or metastatic breast cancers [46]. Surprisingly, this trial failed to demonstrate a benefit in PFS among the B HR+/HER-2-negative cohort (mPFS was 9.3 in both arms, HR 1.00, 95% CI 0.71–1.40), and the OS data are still immature [47]. We do not have results for cohort A, the TNBC population, to see if there is a subgroup of PIK3CA/AKT1/PTEN-altered TNBC that would derive benefits from the combination of ipatasertib and paclitaxel [48].

There are other trials that are continuing to evaluate roles of other AKT inhibitor combinations as therapeutic options for mTNBC. Currently, a phase III trial (NCT04177108) is currently evaluating the efficacy and safety of ipatasertib in combination with atezolizumab and paclitaxel as a first-line therapy among locally advanced or metastatic TNBC [49]. The PATHFINDER trial (NCT04464174), looking at a combination of ipataserib and non-taxane chemotherapy (capecitabine, eribulin, carboplatin and gemcitabine), is also underway [50]. Capivasertib, another AKT inhibitor, is also being evaluated for efficacy and safety when combined with paclitaxel in the CAPItello-290 phase III double-blind randomized study (NCT03997123) [51]. Additional clinical trials have adopted a model of biomarker-driven studies targeting patients with TNBC and a PIK3CA mutation or PTEN loss in order to enrich the biological groups who seem to derive the most benefit. One of these such trials is EPIK-B3 (NCT04251533), a phase III randomized, double-blind, placebo-controlled trial that is currently open and evaluating the safety and effectiveness of alpelisib in combination with nab-paclitaxel among advanced TNBC with a PIK3CA mutation or PTEN loss [52].

### 2.7. Androgen-Targeted Therapy

The comprehensive molecular analyses of breast cancer tumours have demonstrated a subset of TNBC resembling molecular apocrine or luminal androgen receptor (LAR) tumours with androgen receptor activation and its downstream effects [53]. This subset of TNBC has hormone-mediated signalling via androgen receptors. It is hypothesized that the androgen receptor plays a role as an oncogene in TNBC, mediating tumour cell growth, contrary to its anti-oestrogenic and growth-inhibitory influence in ER+ breast cancer [54]. Although they may follow a more indolent course in contrast to other TNBC subtypes, response to traditional chemotherapy is often limited. The AR receptor has therefore become a potential therapeutic target in androgen-expressing TNBC.

This concept was initially studied in the phase II Translational Breast Cancer Research Consortium (TBCRC 011) trial that explored the role of bicalutamide among androgen receptor (AR)-positive, ER- and PR-negative metastatic breast cancer [53]. The primary endpoint was the clinical benefit rate (CBR), and secondary endpoints included the PFS and toxicity. This study identified 12% (*n* = 51) of tumours were AR-expressing breast tumours among 424 patients with ER/PR-negative breast cancer. There were no complete (CR) or partial (PR) responses, but five patients had stable disease for more than 6 months. The study also demonstrated a 6-month CBR of 19% in patients treated with bicalutamide (*n* = 26). The median PFS was 12 weeks, with bicalutamide being well tolerated [53].

This study ultimately led to other phase II clinical trials investigating the anti-tumour activity and safety of other anti-androgen agents such as enzalutamide and abiraterone acetate (a selective inhibitor of CYP17) among AR-positive TNBC patients [55,56]. Enzalutamide was well tolerated and demonstrated clinical activity among patients with advanced AR-positive TNBC in two subgroups evaluated: all enrolled patients (the intent-to-treat (ITT) population) with AR IHC staining >0% and patients with AR IHC staining ≥10% (evaluated subgroup). In this phase II trial, they demonstrated 16- and 24-week CBRs of 35% among the ITT population and 29% among the evaluated subgroup, respectively. The median PFS was 2.9 months among the ITT population and 3.3 months for the evaluated subgroup. Two patients had CR and five patients had PR in this study. The updated analysis showed a median OS of 12.7 months for the ITT population and 17.6 months amongst the evaluable subgroup [55]. Abiraterone acetate plus prednisone also showed a similar trend with a 6-month CBR of 20.0% and a PFS of 2.8 months when evaluated among AR-positive locally advanced or metastatic TNBC in the phase II UCBG 12–1 trial. One patient had CR, and five patients had SD ≥ 6 months and remained on abiraterone acetate plus prednisone [56].

### 2.8. Combined Androgen and PI3K/AKT/mTOR-Pathway-Targeted Agents

A greater understanding of the LAR group has led to the ongoing evolution of therapeutic combinations, including those of AR inhibitors with PI3K inhibitors given the frequent co-amplification of PIK3CA in AR-positive tumours, which is estimated to be as high as 40%. In addition, preclinical work has demonstrated a significant reduction in AR-positive TNBC cell line model growth and viability after treatment with an AR antagonist in combination with PI3K inhibitors [57]. There is currently an ongoing phase IB trial looking at the maximum tolerated dose (MTD), safety, PFS and CBR of alpelisib (PI3K inhibitor) plus enzalutamide among patients with AR-positive and PTEN-positive metastatic breast cancers, including TNBC patients (NCT03207529) [58]. Another phase I/IIB trial showed a trend toward better CBR among AR-positive metastatic TNBC patients when treated with a combination of taselisib (PI3K inhibitor) plus enzalutamide [59]. However, this trial was terminated early in December 2018 after the results of the SANDPIPER trial led to halt in the development of taselisib [59]. This was primarily driven by the fact that only a modest clinical benefit was observed with high discontinuation rates due to toxicity in an ER-positive PIK3CA mutant patient population when given in combination with fulvestrant [60]. Despite the early termination of the taselisib plus enzalutamide trial, there seemed to be an increased clinical benefit. The results of the alpelisib and enzalutamide trial are now awaited.

### 2.9. Combined Androgen Therapy and Cell Cycle Inhibition

The AR-positive TNBC subgroup is thought to have a greater dependence on CDK4/6 phosphorylation, which has sparked interest in studying AR inhibitors in combination with CDK4/6 inhibitors. The safety and effectiveness of palbociclib (CDK 4/6 inhibitor) with bicalutamide is being studied in phase I/II trials among AR-positive metastatic breast cancers (NCT02605486) [61]. The Big Ten Cancer Research Consortium BRE15–024 phase I/II trial is currently looking at the therapeutic combination of ribociclib (CDK 4/6 inhibitor) plus bicalutamide among metastatic or unresectable AR-positive TNBC (NCT03090165) [62].

Finally, immunotherapy as a therapeutic approach among AR-positive metastatic TNBC is also an area of emerging interest. Given that both immunotherapy and AR inhibitors interfere with tumour growth and proliferation, it is thought that they could have synergistic effects leading to better disease control. Currently, a phase II trial is underway, looking at the clinical efficacy and safety of nivolumab (anti-PD-1) in combination with bicalutamide and ipilimumab (anti-cytotoxic T-lymphocyte-associated protein 4, or anit-CTLA4) in HER-2-negative metastatic breast cancers, including AR-positive metastatic TNBC (NCT03650894) [63]. Another phase II trial is looking into the combination of pembrolizumab (PD-1 inhibitor) plus enobosarm (selective androgen receptor modulator) among patients with metastatic AR-positive TNBC (NCT02971761) [64]. We will have to wait for these results to better understand the role of AR inhibition as a therapeutic strategy for the treatment of AR-positive metastatic TNBC.

## 3. Conclusions

TNBC remains a complex disease to treat due to its inter and intra tumour heterogeneity. Unfortunately, the prognosis for patients with advanced TNBC remains poor in contrast to other breast cancer subtypes. Based on current phase III evidence discussed in this review, we recommend tissue tumour tissue testing for PD-L1 and germline BRCA 1/2 testing in all advanced-stage TNBC given the significant therapeutic implications. Based on available data, patients with metastatic PDL-1 positive disease should be offered first-line treatment with pembrolizumab combined with chemotherapy. Among patients with germline BRCA 1/2-mutated TNBC which is PD-L1 negative and does not have a visceral crisis, there is the possibility to consider the first-line use of a PARP inhibitor instead of chemotherapy or otherwise platinum-based chemotherapy if access to PARP inhibitors is limited. For all others, single or combination chemotherapy remains the mainstay of treatment. In the second-line and beyond setting, data support the use of sacituzumab govitecan (SG). Patients who do not meet eligibility criteria or progress beyond the second line should be considered for clinical trials if appropriate or pursue chemotherapy monotherapy. Figure 1 summarizes the treatments for mTNBC based on current phase III evidence available.

Moving forward, a greater understanding of tumour biology will undoubtedly continue to move the field beyond chemotherapeutics. As we progress toward personalized cancer treatment, the integration of tumour genomics, transcriptomics, microenvironment and the immune milieu will be at the core of new developments in the treatment of triple-negative breast cancer. Currently, at our centre, there are multiple ongoing clinical trials evaluating novel combinations in patients with advanced TNBC which could transform the therapeutic landscape. Additionally, our group is evaluating the clinical utility of precision medicine by utilizing tumour genomic alterations to inform oncological treatment. A notable example is the ongoing evaluation of liquid genomic testing to detect tier 1 variants of clinical significance among mTNBC cases that could be used to inform therapeutic approaches or render patients eligible for tumour agnostic clinical trials (PREDiCTI TNBC trial). Another such program is the Personalized Oncogenomics Program (POG) which is utilizing whole-genome and transcriptome analysis to identify potentially informative and actionable molecular alterations, in addition to the evaluation of gene expression signatures and potential predictors of immune response. Notwithstanding the recent advances in mTNBC, the need for a more refined approach to treatment for these patients remains an unmet need that merits future study.

## Figures and Tables

**Table 1:** Phase III clinical trials leading to the current treatment landscape for advanced TNBC.

Trial Identifier	Therapeutic Agent	Class of Agent	Line of Therapy	Phase	Intervention	Key Efficacy Results	Primary Toxicity
Keynote-119 (NCT02555657)	Pembrolizumab	PD-1 inhibitor	≥2nd-line metastatic treatment	III	Pembrolizumab vs. single-agent chemotherapy of physicians’ choice in mTNBC ≥2nd-line metastatic treatment	**PDL-1 CPS ≥10** -mOS 12.7 m vs. 11.6 months (HR 0.78, 95% CI 0.57–1.06) **PDL-1 CPS ≥ 1** -mOS 10.7 months vs. 10.2 months (HR 0.86, 95% CI 0.69–1.06) **Overall Population** -mOS 9.9 vs. 10.8 (HR 0.97, 95% CI 0.82–1.15)	Fatigue, gastrointestinal toxicity, myelosuppression, alopecia, hypothyroidism, hyperthyroidism, pneumonitis, skin reactions, adrenal insufficiency
Keynote-355 (NCT02819518)	Pembrolizumab	PD-1 inhibitor	1st-line treatment	III	Pembrolizumab + chemotherapy (paclitaxel (P) or nab paclitaxel (NP) or carbo/gemcitabine (CG) vs. placebo + chemotherapy (P or NP or CG) in mTNBC as 1st-line treatment	-mPFS 9.7 months vs. 5.6 months (HR 0.65, 95% CI 0.49–0.86) -mOS 23 months vs. 16 months in PDL- positive disease with CPS ≥ 10 (HR 0.73, 95% CI 0.55–0.95)	Fatigue, gastrointestinal toxicity, myelosuppression alopecia, hypothyroidism, hyperthyroidism, pneumonitis, skin reactions, adrenal insufficiency
Impassion-130 (NCT02425891)	Atezolizumab	PD-L1 inhibitor	1st-line treatment	III	Atezolizumab/nab-paclitaxel vs. nab-paclitaxel in mTNBC as 1st-line treatment	**ITT** -mPFS 7.2 months vs. 5.5 (HR 0.80, 95% CI 0.69–0.92) -mOS 21.3 months vs. 17.6 months (HR 0.84, 95% CI 0.69–1.02) **PDL-1 > 1%** -mPFS 7.5 months vs. 5.0 months (HR 0.62, 95% CI 0.49–0.78) -mOS 25 months vs. 15.5 months (HR 0.62, 95% CI 0.45–0.86)	Alopecia, nausea, cough, peripheral neuropathy, neutropenia, pyrexia, hypothyroidism
OlympiAD (NCT02000622)	Olaparib	PARP inhibitor	1st-3rd-line treatment (No more than 2 prior lines of treatment)	III	Olaparib vs. physician’s choice of chemotherapy (capecitabinr, vinorelbine or eribulin) in metastatic germline BRCA 1/2 mutated breast cancer that are HER-2-negative	-mPFS 7 vs. 4.2 months (HR 0.58, 95% CI 0.43–0.80) -mOS 19.3 vs. 17.1 months (HR 0.91, 95% CI 0.66–1.23)	Anaemia, thrombocytopenia, gastrointestinal toxicity
BROCADE 3 (NCT02163694)	Veliparib in combination with carboplatin and paclitaxel	PARP inhibitor	1st-3rd-line treatment (no more than 2 prior lines of treatment)	III	Veliparib in combination with a platinum doublet vs. placebo in combination with platinum in metastatic germline BRCA 1/2 mutated breast cancer that are HER-2-negative	-mPFS 14.5 months vs. 12.6 months (HR 0.71, 95% CI 0.57–0.88)	Myelosuppression
EMBRACA (NCT01945775)	Talazoparib	PARP inhibitor	1st-4th-line treatment (no more than 3 prior lines of treatment)	III	Talazoparib vs. physician choice of chemotherapy (gemcitabine, capecitabine, eribulin or vinorelbine) in metastatic germline BRCA 1/2 mutated breast cancer that are HER-2-negative	-mPFS 8.6 months vs. 5.6 months (HR 0.54, 95% CI 0.41–0.71) -mOS 19.3 months vs. 19.5 months (HR 0.84, 95% CI 0.67–1.07)	Anaemia, thrombocytopenia, gastrointestinal toxicity
Ascent (NCT02574455)	Sacituzumab govitecan	Antibody-drug conjugate targeting trop-2	Post two or more lines of treatment	III	Sacituzumab govitecan vs. choice of chemotherapy (capecitabinr, vinorelbine or eribulin or gemcitabine) in metastatic TNBC	-mPFS 5.6 months vs. 1.7 months (HR 0.41, 95% CI 0.32–0.52) -mOS 12.1 months vs. 6.7 months (HR 0.48, 95% CI 0.38–0.59)	Myelosuppression, gastrointestinal toxicity, fatigue, electrolyte abnormalities, skin changes, infection

**Abbreviations:** Programmed cell death protein 1 (PD-1), programmed death-ligand 1 (PDL-1), median progression-free survival (mPFS), median overall survival (mOS), combined positive score (CPS), intention-to-treat (ITT).

**Table 2:** Novel combination therapeutic treatments actively being evaluated in clinical trials for metastatic TNBC.

Trial Identifier (Clinical Trial.gov)	Class of Agent	Intervention	Phase	Patient Population	Primary (1′) and Key Secondary (2′) Endpoints	Status
**PI3K/AKT/mTOR targeted drug combinations:**						
NCT03337724	AKT inhibitor + chemotherapy	Ipatasertib in combination with paclitaxel vs. paclitaxel	III	Patients with PIK3CA/AKT1/PTEN-altered, locally advanced or metastatic, triple-negative breast cancer or hormone receptor-positive, HER2-negative breast cancer	1′: PFS 2′: ORR, DOR, CBR, OS	Active, not recruiting, Start date: 6 January 2018 Estimate date of completion: 22 December 2022
NCT04177108	AKT inhibitor + anti-PDL1	Ipatasertib in combination with atezolizumab and paclitaxel	III	Locally advanced or metastatic triple-negative breast cancer	1′: PFS, OS 2′: AEs, ORR, DOR, CBR	Active, not recruiting, Start date: 25 November 2019 Estimate date of completion: 10 October 2025
NCT04464174 (PATHFINDER)	AKT inhibitor + chemotherapy	Ipatasertib plus chemotherapy (capecitabine or eribulin or carboplatin plus gemcitabine)	III	Taxane-pretreated, unresectable, locally advanced or metastatic triple-negative breast cancer patients	1′: Safety and tolerability 2′: PFS, TTR, ORR, DOR, CBR, OS	Recruiting Start date: 8 October 2020 Estimated end date: 31 March 2022
NCT03997123 (CAPItello-290)	AKT inhibitor + chemotherapy	Capivasertib + paclitaxel	III	First-line treatment for patients with locally advanced (inoperable) or metastatic TNBC	1′: OS 2′: PFS, ORR, AEs, DOR, CBR	Recruiting Estimated start date: 25 June 2019 Estimated end date: 24 March 2023
NCT04251533	PI3K inhibitor + chemotherapy	Alpelisib in combination with nab-paclitaxel vs. nab-paclitaxel	III	Patients with advanced TNBC with either PIK3CA or PTEN loss without PIK3CA mutation	1′: PFS, ORR 2′: OS, CBR, TTR, DOR	Recruiting Estimated start date: 8 June 2020 Estimated end date: 9 January 2026
**Novel antibody-drug conjugate (ADC) combinations:**						
NCT03310957 (SGNLVA-002)	ADC + PD-1 inhibitor	SGN-LIV1A (iadiratuzumab vedotin) plus pembrolizumab	IB/II	First-line treatment of patients with unresectable locally advanced or metastatic triple-negative breast cancer	1′: ORR, AEs, lab abnormalities 2′: DOR, PFS, OS	Recruiting Estimated start date: 27 February 2018 Estimated End date: 30 April 2023
NCT03424005 (Morpheus-TNBC)	ADC + PD-1 inhibitor	Multiple immunotherapy-based treatment combinations including ADC combinations (atezolizumab + sacituzumab govitecan and atezolizumab + SGN-LIV1A)	I/IIB	Metastatic or inoperable locally advanced TNBC	1′: ORR, AEs 2′: PFS, DCR, OS, DOR	Recruiting Estimated start date: 2 April 2018 Estimated End date: 30 March 2023
NCT05143229 (ASSET)	PI3K inhibitor + ADC	Alpelisib plus sacituzumab govitecan	I	Metastatic or locally recurrent HER2-negative breast cancer including TNBC	1′: RP2D 2′: ORR, pharmacokinetics	Recruiting Estimated start date: 28 March 2022 Estimated end date: June 2024
NCT04602117 (ISPY-P1.01)	ADC + chemotherapy	Vic-trastuzumab duocarmazine (SYD985) + weekly paclitaxel	I	Evaluating the safety of ADC+ chemotherapy in patients with metastatic cancer including TNBC	1′: AEs, CBR, ORR 2′: PFS, DOR	Recruiting Estimated start date: 28 July 2021 Estimated end date: 1 December 2022
NCT04556773 (DESTINY-Breast08)	ADC + other anti-cancer agents (chemotherapy, immunotherapy + chemotherapy, AKT inhibitor, aromatase inhibitor, or oestrogen receptor antagonist)	Trastuzumab deruxtecan (T-DXd) in combination with other anti-cancer agents (capecitabine, durvalumab and paclitaxel, capivasertib, anastrozole or fulvestrant)	IB	Metastatic HER2-low breast cancer (including TNBC)	1′: AEs, SAEs 2′: ORR, PFS, DOR, OS	Recruiting Estimated start date: 17 December 2020 Estimated end date: 28 August 2023
NCT04039230	ADC + PARP inhibitor	sacituzumab govitecan plus talazoparib	IB/II	Metastatic TNBC	1′: Dose-limiting toxicity 2′: DOR, TTR, PFS, OS	Recruiting Estimated start date: 9 October 2019 Estimated end date: 31 October 2024
NCT03729596	Anti-B7-H3 antibody-drug conjugate alone and + Anti-PD-1 antibody	MGC018 alone and in combination with retifanlimab	I/II	Advanced solid tumours including TNBC	1′: AEs, MTD 2′: Preliminary anti-tumour activity, patient outcome, radiographic PFS	Recruiting Estimated start date: 21 November 2018 Estimated end date: May 2023
NCT03504488	ROR2-targeted ADC alone and + PD-1 inhibitor	CAB-ROR2-ADC alone and plus PD-1 inhibitor	I/II	Locally advanced unresectable or metastatic solid tumour including TNBC	1′: ORR, pharmacokinetics 2′: DOR, OR, DCR, TTR, PFS, OS	Recruiting Estimated start date: 27 June 2018 Estimated end date: 30 June 2023
NCT04468061	ADC + PD-1 inhibitor	Sacituzumab govitecan with or without pembrolizumab	II	PD-L1-negative metastatic triple negative breast cancer	1′: PFS 2′: ORR, CBR, DOR, TTP, TTOR, OS	Recruiting Estimated start date: 20 July 2020 Estimated end date: 1 April 2027
**Novel androgen receptor (AR) inhibitor combinations:**						
NCT03207529	PI3K inhibitor + AR inhibitor	Alpelisib plus enzalutamide	IB	Patients with androgen receptor (AR)- positive and PTEN-positive metastatic breast cancer (including TNBC)	1′: MTD 2′: AEs, PFS, CBR	Recruiting Estimated start date: 7 June 2019 Estimated end date: 31 December 2020
NCT03090165 (Big Ten Cancer Research Consortium BRE15–024)	CDK 4/6 inhibitor + AR inhibitor	Ribociclib plus bicalutamide	I/II	Metastatic or unresectable AR+ triple-negative breast cancer (TNBC)-AR-positive defined as IHC staining of >0%	1′: phase I Max tolerated dose, CBR 2′: ORR, DOR, AEs, PFS, OS	Active, not recruiting Estimated start date: 2 March 2017 Estimated end date: September 2024
NCT02605486	CDK 4/6 inhibitor + AR inhibitor	Palbociclib plus bicalutamide	I/II	AR(+) metastatic breast cancer (MBC) including TNBC in phase I part	1′: RP2D, PFS 2′: ORR, CBR, PFS after 1 year, AEs	Active, not recruiting Estimated start date: 11 November 2015 Estimated end date: November 2023
NCT03650894	Immunotherapy + AR inhibitor	Nivolumab combined with ipilimumab plus bicalutamide	II	Metastatic HER2-negative breast cancer—TNBCs were allowed in the study as long they had confirmation of androgen receptor (AR) positivity at screening	1′: CBR 2′: ORR, PFS, OS	Recruiting Estimated start date: 3 April 2019 Estimated end date: April 2025
NCT02971761	Immunotherapy + selective androgen receptor modulator (SARM)	Pembrolizumab plus enobosarm	II	Patients with metastatic androgen receptor (AR)-positive triple-negative breast cancer (TNBC)	1′: AEs, RR, DLT 2′: CBR, EFS, TTF, PFS, OS	Active, not recruiting Estimated start date: 1 June 2017 Estimated end date: 3 November 2021

**Abbreviations:** Progression-free survival (PFS), overall survival (OS), response rate (RR), percentage of participants with adverse events (AEs), serious adverse events (SAEs), overall response rate (ORR), duration of response (DOR), clinical benefit rate (CBR), disease control rate (DCR), maximum tolerated dose (MTD), recommended phase II dose (RP2D), best overall response (OR), event-free survival (EFS), time-to-treatment failure (TTF), time to objective response (TTOR) and time to response (TTR).
